# Ultraviolet‐C Light Emitter as a Method for Reducing Bacterial Contamination of Ophthalmic Instruments

**DOI:** 10.1155/joph/6924280

**Published:** 2026-09-27

**Authors:** Raul Velez-Montoya, K. Carolina Franco-Ramirez, Abril T. Vargas-Fernández, Jesus E. Arreola-Martinez, J. Armando Cruz-Cervantes, Virginia Vanzzini-Zago, Jans Fromow-Guerra

**Affiliations:** ^1^ Retina Department, Asociación para Evitar la Ceguera en México IAP, Mexico City 04030, Mexico, apec.org.mx; ^2^ Microbiology Department, Asociación para Evitar la Ceguera en México IAP, Mexico City 04030, Mexico, apec.org.mx

**Keywords:** decontamination, endophthalmitis, in-office procedures, intravitreal, ultraviolet type C light

## Abstract

**Background:**

To assess the efficacy of an ultraviolet light type C (UVC) emitter in reducing bacterial load in common surgical equipment used during in‐office ophthalmological procedures.

**Methods:**

We used 7 steam‐sterilized lid speculums. The first one was the negative control. The second was left unprotected in a high‐traffic area for 24 h before being cultivated. Lid speculums 3 to 7 were contaminated with a strain of *Staphylococcus epidermidis*, recovered from an active case of postoperative endophthalmitis. The third one was cultivated immediately after contamination. Lid speculums 4 to 7 were exposed to UVC for 30 s, 2, 5, and 10 min, respectively (continuous 254 nm UVC, irradiance > 160 mW/cm^2^). All speculums were cultivated on Mueller–Hinton agar plates for 48 h (≈21% O_2_). Differences in bacterial load, observed colonies, and colony‐forming units were assessed with a Kruskal–Wallis test. All experiments were done in triplicate.

**Results:**

We analyzed 27 bacterial cultures. After 24 h, lid speculum 2 showed growth of a single colony of *Sphingomonas paucimobilis.* After 30 s of UVC exposure, bacterial growth was 108 ± 7.2 CFU (bacterial load: 3240 ± 214.7). Bacterial growth decreased progressively with increasing UVC exposure, reaching no detectable growth after 5 min, representing a 100% reduction (*p* < 0.01). A second culture obtained from lid speculum 2 and the positive control after additional 5 min of UVC exposure also showed no bacterial growth.

**Conclusions:**

This exploratory laboratory study suggests that, after 5 min of UVC exposure using a commercially available emitter, no recoverable bacteria were detected on lid speculums under these experimental conditions. Although spores, fungi, and viruses were not evaluated, these findings suggest that UVC irradiation may be a feasible approach for reducing bacterial contamination of reusable instruments during in‐office ophthalmic procedures.

## 1. Introduction

In ophthalmology, minor in‐office procedures are common. While some of these procedures, such as examinations using a Goldmann applanation tonometer, diagnostic contact lenses (three‐mirror, wide‐field posterior fundus examination, etc.), or a meibomian gland expressor, are noninvasive, others are invasive and may involve the use of needles, lid speculums, surgical blades, forceps, and/or scissors [1–6]. These procedures, although very safe in expert hands, carry an increased risk of complications.

Ocular infections secondary to in‐office procedures can take many forms, from mild and transient, such as bacterial or viral blepharitis contracted during an examination, to severe and sight‐threatening, such as ulcerative keratitis secondary to corneal suture removal or endophthalmitis postintravitreal injection [6–10]. To decrease the risk of infection, strict adherence to sterile techniques is strongly advised during in‐office procedures. This requires using chemical disinfectants for eyelids and conjunctiva, sterile drapes, face masks, and gloves, when necessary, as well as sterile surgical instruments for more invasive procedures [11–13].

Maintaining the sterility of surgical equipment in an in‐office environment can be challenging compared to in a hospital setting. Traditional sterilization methods include physical methods such as heat, radiation, gas (ethylene oxide), and various chemical agents [14, 15]. Although these methods are effective, well‐validated, widely accepted, and remain the regulatory standard in hospital settings, they also have significant drawbacks and challenges in an in‐office setting [16, 17].

The operational costs and the need for specialized equipment and maintenance, and time‐consuming protocols to ensure the effective elimination of pathogens, spores, molds, and viruses, may lead to improper adherence to sterile techniques [15, 18]. Moreover, improper storage or prolonged shelf time of properly sterilized equipment in low‐volume clinics and offices may compromise sterility. Administrative errors in the control of expiration dates and improper handling of chemical products may also impact efficiency [10, 15, 18]. Nevertheless, it is important to distinguish between sterilization and disinfection. Sterilization refers to the complete elimination of all microorganisms, including spores, and remains the hospital and surgical standard. Disinfection involves reducing pathogenic microorganisms to levels considered safe, and is often a more practical protocol for in‐office procedures [19].

Ultraviolet light type C (UVC) has been used for surface, water, and medical equipment disinfection due to its effectiveness and low cost [20–22]. Its specific wavelength causes irreversible damage to ribonucleic acid (RNA) and deoxyribonucleic acid (DNA) bases, interrupting cellular replication and inducing oxidative damage [23]. The efficacy of UVC depends on several variables, including pathogen classification, light intensity, emission pattern (continuous vs. pulsed), wavelength, and exposure duration [22, 23]. Furthermore, previous studies have demonstrated a dose‐dependent reduction in bacterial contamination of medical devices and surgical instruments with increasing UVC exposure time and irradiance, underscoring the importance of dose–response relationship in clinical applications [24, 25].

The application of UVC for the disinfection of ophthalmic surgical instruments used during in‐office procedures has not been previously reported [22]. This method’s unique advantages of speed and low cost, combined with its safety and efficacy, suggest that implementing UVC disinfection may improve physician adherence to sterility protocols during in‐office procedures and streamline processes in the office.

The objective of the current study was to assess the efficacy of a commercially available UVC emitter in reducing bacterial contamination of ophthalmic surgical instruments commonly used during intravitreal injections (lid speculum). By quantifying bacterial load, colony counts, and colony‐forming units (CFU) under different exposure times, we aimed to assess the feasibility of this approach for in‐office disinfection procedures. We hypothesize that increasing UVC exposure time would result in a dose‐dependent reduction in bacterial contamination.

## 2. Methods

This experimental laboratory investigation was conducted in accordance with good clinical and laboratory practice guidelines. In compliance with local regulations, the Institutional Review Board reviewed and approved the experiment because the bacterial isolate used in this study was obtained from a human clinical specimen, although no human subjects were directly enrolled. Generative artificial intelligence (AI) was not employed in the development of the manuscript’s conceptual content. However, AI‐assisted tools were used to support spelling correction, grammar refinement, and improvements in readability.

We used 7 lid speculums that had recently been sterilized by a steam sterilizer at the hospital’s equipment center. The first lid speculum served as a negative control. The second lid speculum was used to simulate improper handling and a lack of adherence to sterile techniques before intravitreal injections. For this, after the sterile seal was broken, we left the speculum exposed to the environment in a high‐traffic area of the retina service clinic for 24 h before the experiment. The third lid speculum served as a positive control. The fourth to the seventh lid speculums served as the experimental group.

For this experiment, we used a clinical strain of *Staphylococcus epidermidis* (coagulase‐negative staphylococci [CoNS]) isolated from a vitreous sample obtained from a patient with active postoperative endophthalmitis. The isolate was stored under appropriate conditions and phenotypically characterized using Gram staining, catalase and coagulase testing, and the VITEK 2 identification system (bioMérieux, Marcy‐l’Étoile, France). No genotypic characterization was performed. Under a laminar flow hood and using a sterile inoculating loop, the isolate was streaked in a Z pattern onto a sterile Mueller–Hinton agar plate. The plate was incubated at 37°C for 48 h under aerobic conditions (≈21% O_2_ and 4% CO_2_). Culture viability was confirmed by visible bacterial growth. The identity of the isolate was verified by the presence of Gram‐positive cocci arranged in clusters, positive catalase and negative coagulase reactions, and by confirmation with the VITEK 2 identification system.

All experiments were conducted under a laminar flow hood, and bacterial suspensions were prepared using the previously described strain of CoNS. All cultures were incubated for 48 h at 37°C under aerobic conditions. A negative culture was defined as the absence of observable bacterial growth in the culture medium, confirmed by a negative Gram stain control of the same culture. Colony quantification was performed through direct visualization using a FE‐500 colony counter (Felisa, Fabricantes Feligneo SA, Mexico).

The first lid speculum (negative control) was cultivated immediately after the sterile seal was broken. In this case, we placed the lid speculum in 3 mL of sterile balanced salt solution (BSS) for 1 min. Then, using a micropipette, we collected 1 mL and inoculated a sterile Mueller–Hinton agar plate, allowing the solution to homogenize over the surface and penetrate the culture media. The second lid speculum, mimicking mishandled equipment and lack of adherence to sterile techniques, was processed with the same procedure after 24 h of unrestricted environmental exposure. The third lid speculum (positive control) was processed as follows: The lid speculum was submerged in a 0.5‐McFarland Standard CoNS suspension (≈1.5 × 10^8 CFU/mL) for 1 min immediately after the sterile seal was broken. The lid speculum was left to dry over a sterile Petri dish lid for 15 min and then lavaged with 3 mL of sterile BSS for 1 min. A sample of the lavage solution underwent decimal dilution (1:10; 200 μL of lavage solution plus 1800 μL of sterile BSS), and using a micropipette, we inoculated a sterile Mueller–Hinton agar plate with 1 mL of the mixture, allowing the solution to homogenize over the surface and penetrate the culture media.

The fourth to seventh lid speculums were sequentially processed as follows: The lid speculum was submerged in a 0.5‐McFarland Standard CoNS suspension for 1 min immediately after the sterile seal was broken. The lid speculum was left to dry over a sterile Petri dish lid for 15 min and then placed alone inside the loading bay of a UVC emitter device (Ennva® products, China), where it was exposed to UVC radiation for a set duration.

The fourth speculum was exposed to UVC radiation for 30 s, the fifth for 2 min, the sixth for 5 min, and the seventh for 10 min (Figure 1). These intervals were selected pragmatically to represent short, intermediate, and extended exposure that could be realistically applied in clinical practice, and to allow assessment of progressive dose–response effect [20, 24, 25]. All speculums were irradiated using the same parameters: continuous UVC irradiation with a wavelength of 254 nm emitted by a high‐quality UVC quartz lamp with an irradiance > 160 mW/cm^2^, potency 6 W (110–120 V), and lamp current 3 mA. The speculums were placed in the center of the box on a stainless steel grid positioned 1 cm above the UVC lamp emitter. Each speculum was maintained in the same horizontal orientation on the grid, with an average distance of 5 cm from the lateral walls and 5.2 cm from the lid of the device. The UVC emitter device was housed within the bay of the laminar flow hood and remained inside for the duration of the experiment. The device diagram is shown in Figure 2. The UVC emitter device was handled and operated in accordance with the safety guidelines recommended by the National Environment Agency of Singapore. These included clear labeling of the room where the experiment was conducted and the use of personal protective equipment by all operators and researchers (UV‐resistant eyewear and protective clothing for the arms and other exposed skin areas) [26].

**FIGURE 1:**
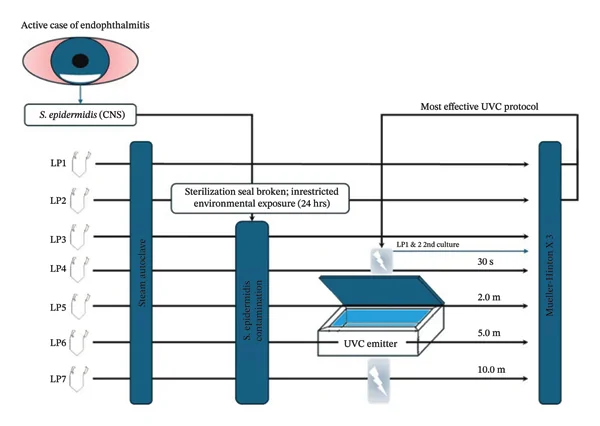
Experiment summary. All experiment procedures were done in triplicate. Experiments with the lid speculums 4 to 7 were done first to detect the most effective UVC protocol. Lid speculums 2 and 3 were done last. LP, lid speculum; UVC, ultraviolet type C light; s, seconds; m, minutes.

**FIGURE 2:**
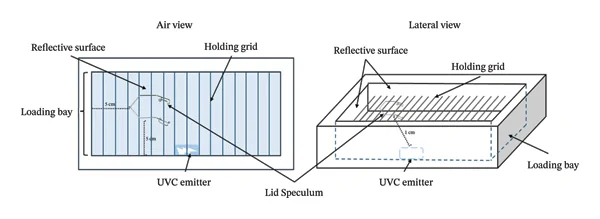
Device characteristics. Loading bay: length: 25.9 cm; deep: 14.7 cm; height: 8.6 cm. In this depiction, the closing lid has been removed from the diagram, which has a hermetic seal. The power cord and on/off switch are not shown. All internal walls of the loading bay had reflecting mirrors, including on the closing lid. UVC, ultraviolet type C light.

After radiation, each speculum was lavaged with 3 mL of sterile BSS for 1 min. Then, using a micropipette, we collected 1 mL and inoculated a sterile Mueller–Hinton agar plate, allowing the solution to homogenize over the surface and penetrate the culture media. All inoculated Mueller–Hinton agar plates were incubated at 37°C for 48 h under aerobic conditions (21% O_2_ and 4% CO_2_) to assess the bacterial load, number of colonies, and CFU count on each plate. Bacterial load was defined as the quantitative measure derived from colony counts and CFU/mL calculations, obtained through decimal dilution and direct visualization using a FE‐500 colony counter [27]. All experiments were repeated in triplicate.

Finally, after the bacterial load, number of colonies, and CFU count from the second and third lid speculums (representing mishandled equipment and lack of adherence to sterile techniques and the positive control, respectively) were assessed, we placed the speculums separately inside the UVC emitter device, using the most efficient time interval observed during the experiment. Then, the culture process was repeated as described earlier.

Statistical analysis was performed via an Excel spreadsheet (Excel 2010; Microsoft Corp., Redmond, WA) with XLSTAT v18.06 (Addinsoft, New York, NY). Continuous variables were presented as means and standard deviations. Comparison between results of each lid speculum bacterial count was done with a nonparametric analysis of variance (Kruskal–Wallis test), linear regression was employed to analyze the relationship between variables and UVC exposure time, with an alpha value of 0.05 indicating statistical significance for both analyses.

## 3. Results

We analyzed 27 bacterial cultures from 7 lid speculums, including 6 additional cultures from the second and third lid speculums (the experiment was performed in triplicate). No bacterial growth was observed in any of the cultures from the first lid speculum (negative control).

One of three cultures from the second lid speculum (mishandled equipment and lack of adherence to sterile techniques) was negative. The other two cultures produced small yellow bacterial colonies with convex and smooth edges. Gram staining revealed a low‐motility Gram‐negative bacillus, which was classified as *S. paucimobilis* by VITEK 2 (Figure 3).

**FIGURE 3:**
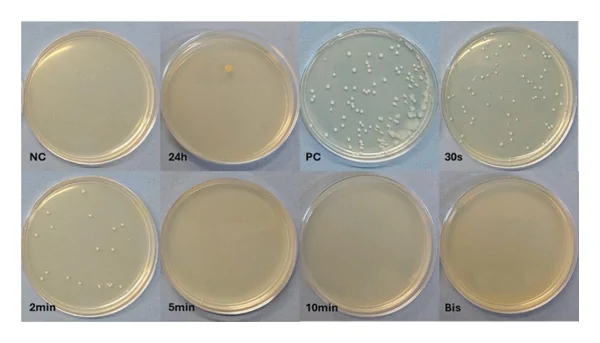
Culture results. NC: Lid speculum #1, negative control. 24 h: Lid speculum #2, mishandled equipment. PC: Lid speculum #3, positive control. 30 s: Lid speculum #4, 30 s UVC exposure. 2 min: Lid speculum #5, 2 min UVC exposure. 5 min: Lid speculum #6, 5 min UVC exposure. 10 min: Lid speculum #7, 10 min of exposure. Bis: Lid speculums 2 and 3 after 5 min of UVC exposure.

Table 1 summarizes the bacterial load, number of colonies, and CFU count observed in cultures from the second to seventh lid speculums. All bacterial growths (except those on the second lid speculum) were classified as catalase‐positive, coagulase‐negative, Gram‐positive cocci and characterized as CoNS by VITEK 2. There was a statistically significant difference in the bacterial load, number of colonies, and CFU count among the cultures (Figure 3). The positive control yielded the greatest bacterial load, number of colonies, and CFU count (*p* < 0.01). All parameters decreased as the duration of UVC exposure increased. All counts and bacterial growth were negative (zero) at 5 and 10 min of UVC exposure, respectively (the sixth and seventh lid speculums). All six additional cultures from the second and third lid speculums were negative after 5 min of UVC exposure. No *S. paucimobilis* was observed in the Gram stains or in the second set of cultures from the second lid speculum.

**TABLE 1:** This table summarizes the overall results from all bacterial and colonies count.

Lid speculum	Colonies	CFU	Bacterial load
#1 Negative control	0	0	0
#2 Mishandle (24 h)	0.5 ± 0.2 (95% CI: 0–1)	0.5 ± 0.2 (95% CI: 0–1)	1.5 ± 0.7 (95% CI: 0–3.01)
#3 Positive control	108 ± 9.8 (95% CI: 91.7–124.2)	1080 ± 71.6 (95% CI: 917.9–1242.09)	3240 ± 214.7 (95% CI: 2753–3726.2)
#4 UVC 30 s	39 ± 9.8 (95% CI: 16.7–61.2)	39 ± 9.8 (95% CI: 16.7–62)	117 ± 229.5 (95% CI: 50.1–183.8)
#5 UVC 2 min	17 ± 3.1 (95% CI: 9.9–24.09)	17 ± 3.0 (95% CI: 9.9–25)	51 ± 9.4 (95% CI: 29.7–72.2)
#6 UVC 5 min	0	0	0
#7 UVC 10 min	0	0	0
#2 and #3 Bis (5 min)	0	0	0

*Note:* Results show that bacterial growth decreased progressively with increasing UVC exposure, reaching complete inhibition (100% reduction; nondetectable growth) after 5 min. #2 and #3 Bis are the results of the second bacterial count from the lid speculums 2 and 3 after 5 min of UVC exposure. UVC: ultraviolet light type C.

Abbreviations: CFU, colony‐forming units; CI, confidence interval.

Linear regression confirmed a significant inverse dose relationship between UVC exposure time and all tested variables. Increasing UVC exposure was associated with a progressive reduction in colony counts (*β* = −0.125, 95% CI: −0.179 to −0.075, *p* < 0.01), with exposure time accounting for 42.7% of the observed variability (*R* ^2^ = 0.427). Likewise, CFU counts declined significantly with increasing UVC exposure (*β* = −0.999, 95% CI: −1.56 to 0.42; *p* = 0.0012), with exposure time explaining 27.4% of the variability (*R* ^2^ = 0.274). Similarly, bacterial load decreased significantly as exposure time increased, with each additional second of UVC exposure resulting in a mean reduction of 2.82 bacteria (*β* = −2.82, 95% CI: −4.59 to −1.04; *p* = 0.0028). UVC exposure time accounted for 24% of the variability in bacterial load (*R* ^2^ = 0.240) (Figure 4).

**FIGURE 4:**
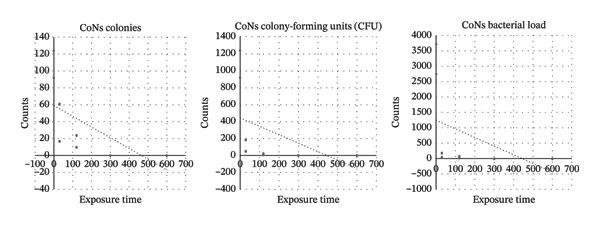
Scatterplot and regression line of the three main variables. The plot shows an inverse relationship between UVC exposure time and the counts of colonies, colony‐forming units, and bacterial load. After 5 min of UVC exposure, no bacteria were observed. CoNS, coagulase‐negative staphylococcus.

## 4. Discussion

UVC radiation is an effective sterilization method for water, air, and inanimate surfaces such as hospital beds/rooms, surgical rooms, ambulances, personal protective equipment, surgical microscopes, surgical instruments, and prosthetics [23, 28, 29]. Its germicidal effect at a peak wavelength of 245 nm is attributed to its ability to disrupt the nucleic acids and proteins of microorganisms, leading to inactivation and loss of viability [23, 30]. This radiation destabilizes the double bonds between carbon atoms in purines and pyrimidines in nucleic acids, promoting the formation of cyclobutyl pyrimidine dimers (CPDs) and pyrimidine (6–4) pyrimidone photoproducts (6‐4PPs), successfully interrupting DNA replication and the cell cycle [30, 31]. This fundamental cell damage ultimately kills bacteria and fungi and induces the inactivation of viruses and molds [23, 29]. Studies on the effectiveness of various UVC sterilization methods have demonstrated an ability to deactivate airborne particles, good germicidal effects against most ESKAPE‐E pathogens (*Enterococcus faecium*, *Staphylococcus aureus*, *Klebsiella pneumoniae*, *Acinetobacter baumannii*, *Pseudomonas aeruginosa*, *Enterobacter* spp., and *Escherichia coli*), and a lack of significant difference from steam autoclaving method in terms of CFU count reduction while maintaining the structural integrity of metallic surfaces [32–34].

In‐office procedures are a common part of everyday ophthalmological practice. A comprehensive ophthalmological examination may include exploration maneuvers that use specialized equipment that comes very close to or in contact with the ocular surface [5, 35]. In special situations, physicians may need to perform even more invasive procedures that require probes, scissors, forceps, lid speculums, and/or needles [6]. Such procedures may involve lacrimal duct instrumentation, suture removal, chalazion treatment, intravitreal injections, pneumoretinopexy, and slit‐lamp air‐fluid exchange [2, 6, 36–38]. Equipment that has been improperly handled, cleaned, or sterilized may lead to the propagation of bacterial or viral infections of varying degrees of severity and complications [9].

Ulcerative keratitis and endophthalmitis may be the most feared infectious complications related to in‐office ophthalmic procedures. The latter is usually associated with invasive procedures such as intravitreal injections [8]. Even though there is no standardized technique for intravitreal injections, consensus among specialists suggests that, among other recommendations, the use of a lid speculum during injection may decrease the risk of eyelash contact with the needle and improve scleral exposure [12, 13]. Although there is no definite evidence that this is completely necessary or better than manual retraction of the eyelid in reducing the risk of postinjection endophthalmitis, if a physician chooses to use the former, the use of sterile equipment is always preferred and encouraged [11–13].

In‐office sterilization methods are usually limited to applications of heat and liquid chemicals. Larger offices and medical groups may also employ steam autoclaves or ethylene oxide gas. While these methods are highly effective in achieving sterility, they entail additional operational challenges and costs, and require specialized personnel and adequate facilities [10, 39]. Consequently, in‐office sterilization remains difficult and may compromise proper adherence to sterile technique [15, 18].

In this study, we assessed the efficacy of different UVC radiation protocols in reducing the viable bacterial load on lid speculums purposely contaminated with CoNS. We attempted to mimic the real‐life scenario of a lid speculum being mishandled by leaving it exposed without any particular protection in a high‐traffic area of our clinic. We chose to use a strain of *S. epidermidis* because it is the most commonly associated pathogen with postintravitreal endophthalmitis injection. The strain was first recovered from a patient with active endophthalmitis and grown in culture to ensure that the bacteria were sufficiently virulent to cause infection. Our results suggested that longer exposure to UVC may lead to a significant reduction in the bacterial load, number of colonies, and CFU count observed in culture. Even the lid speculum that presented the most bacteria (third lid speculum, positive control) was negative after 5 min of exposure to UVC. These findings could change daily ophthalmological practice. By introducing a fast and efficient decontamination method, daily examinations and in‐office procedures can be performed with safer and cleaner instruments, without interrupting patient flow or increasing the complexity of in‐office processes, with the added benefit of potentially reducing the risk of infection. Moreover, it may hypothetically also represent significant long‐term savings. For example, depending on local energy cost (watts/hour), 5 min of exposure with a 6‐W UVC emitter may correspond to an expense of ≈0.06 to ≈0.15 USD. Thus, a high‐flow clinic administering an average of 40 intravitreal injections per day could have all instruments decontaminated for ≈6.12 USD. The speed of the process may also reduce the need for an ample stock of instruments because a small number of instruments can be cleaned and decontaminated multiple times, or on demand, without damaging the instruments. Finally, the substitution of chemicals, gas, and heavy equipment for an energy‐efficient method could lead to a reduction in the practice’s carbon footprint.

In addition to its small sample size, limited experimental groups (speculums), and few replications, our study presents several limitations. Although we employed a nonselective, nondifferential growth medium (Mueller–Hinton agar) suitable for the isolation of CoNS, it may be less suitable for cultivating slow‐growing bacteria, anaerobic bacteria, or other fastidious organisms. This may explain the low bacterial counts observed on the second lid speculum, and the results of this study may not necessarily reflect the effect of UVC decontamination on the broader array of bacteria that could be isolated from a mishandled instrument. Moreover, the growth medium employed was not conducive to fungal or viral growth, nor did we include such agents in our experiment. In addition, we did not test the device with biofilm‐forming bacteria. These factors limit our observations to Gram‐positive bacteria only, specifically *S. epidermidis*. Although this organism is responsible for 60%–70% of endophthalmitis cases secondary to intravitreal injections, it is not possible to determine the effect of UVC irradiation on other bacteria, fungi, viruses, molds, or biofilm‐producing microorganisms based on these results. Furthermore, we did not include comparison with established sterilization methods. This restricted scope substantially limits the applicability of our findings, and results should be interpreted within the narrow context of the tested organism before considering broader clinical implementation.

Although the DNA damage induced by UVC seemed to inactivate the bacteria in our experiment, our design proved only short‐term efficacy by inhibiting bacterial replication and growth in culture media. Our results suggest that instruments used for an in‐office procedure can be employed immediately after irradiation, with the expectation of achieving a certain degree of decontamination. However, bacteria that survive the initial exposure and have DNA‐repairing enzymes may regain viability and virulence over time [40]. Therefore, equipment from long‐term storage may require further UVC irradiation before use, even if it was decontaminated before storage. Readers should also be aware that the peak sensitivity for bacterial DNA damage occurs at 265 nm. Thus, employing a commercially available device that emits at a slightly shorter wavelength will affect the energy requirements needed to achieve the same expected effect. However, even with this limitation, our results demonstrate that a reduction in bacterial load can be achieved without requiring excessively long exposure times [41].

The decontamination effect peaks and is limited to the surface of the intended object. Therefore, it works best on solid objects with smooth and simple surfaces. Objects with complex shapes, with self‐casting shadows, with inner cavities, or with damaged or cracked surfaces could create areas shielded from UVC, decreasing its efficacy. UVC light has also been shown that it could be reactive with certain chemicals and hydrogels; hence, instruments with plastic parts or that have been chemically treated to reduce glare and reflections, such as bio and slit‐lamp lenses, could sustain damage [22, 34, 42].

Another limitation is that, although the UVC emitter was used according to the manufacturer’s instructions, the emitted wavelength and irradiance were not independently verified. Hence, the delivered UVC fluence could not be quantified, and the findings are therefore reported as exposure times under the experimental conditions in this study. Since this device is not medical‐grade equipment, some variability in emission could be expected. This variability may reduce the effectiveness of bacterial load reduction or alter the time required to replicate our results.

Finally, our study did not use advanced molecular techniques to prove bacterial DNA damage. We suspect that the sudden loss of growth capability may be proof of significant structural DNA damage, especially considering that the bacteria were initially recovered from an active infection site and successfully grew in culture before the experiment. Nevertheless, a second confirmatory test could have increased the level of confidence in our results and further supported the use of UVC as a decontamination method.

In summary, the exposure of surgical instruments (such as a lid speculum) commonly used during ophthalmological in‐office procedures for 5 or more minutes to 254 nm UVC with an irradiance of 160 mW/cm^2^ potentially decreases the bacterial load and severely impairs the ability of bacteria to grow in culture. The adoption of this decontamination method for surgical and exploratory instruments could lead to a decrease in the incidence of in‐office‐transmitted infections.

## Funding

No funding was received for this manuscript.

## Disclosure

The current manuscript has never been published. A preliminary version was presented as a poster during the ARVO annual meeting in Salt Lake City, 2025. The authors do not have any economic, proprietary, or financial interest to disclose in the publication of this paper. There were no funds allocated to the realization of this research. The authors state that they have full control of all primary data and agree to allow *Journal of Ophthalmology* to review their data upon request.

## Ethics Statement

This in vitro study did not involve human participants, human data or tissue, or live animals. Therefore, institutional ethics committee approval and informed consent were not required. Per local regulations, all research projects are reviewed and approved by the Asociación para Evitar la Ceguera Internal Review Board, which is regulated and supervised by the Mexican Ministry of Health (Secretaría de Salud) and the National Bioethics Commission (CONBIOÉTICA). IRB approval number: RE‐24‐05.

## Conflicts of Interest

The authors declare no conflicts of interest.

## Data Availability

The data that support the findings of this study are available from the corresponding author upon reasonable request.
